# Supplementary material for: SMRT genome assembly corrects reference errors, resolving the genetic basis of virulence in Mycobacterium tuberculosis
Source: BMC Genomics. 2017 Apr 17;18:302. doi: 10.1186/s12864-017-3687-5 (PMC5393005; doi:10.1186/s12864-017-3687-5)
Supplement: Supplementary file 3 — Expanded Discussion of Virulence Attenuation Mechanisms in M. tuberculosis H37Ra. (PDF 136 kb) [file 12864_2017_3687_MOESM3_ESM.pdf]

Expanded Discussion of H37Ra-specific Variants

Background on Genes

Supplementary Table 1 shows the breakdown of the genes affected by reported "H37Ra-specific" variants (Table 1) by their confirmation status and TubercuList functional categories [1]. Of the 18 genes adjusted or supported, 7 functional categories are represented. PE_PPE (6 genes) and intermediary metabolism (3 genes) comprise half of these genes. This updated polymorphism distribution reduces the set of genomic factors potentially causal to the phenotype of H37Ra, but does not preclude the possibility of multiple distinct processes contributing.

Below, the published information on each of the genes with variants reported as unique to H37RaJH with respect to H37Ra are reviewed in the context of our updated assembly. They are subdivided by TubercuList functional category. Several of the variants in H37RaJH with respect to H37Rv supported by our assembly (Table 1c, Supplementary Table 1), particularly hypothetical genes, are not well-studied. These genes, though they should not be excluded from consideration for conferring the attenuated virulence phenotype to H37Ra, are not discussed at length in the following subsections.

Regulatory and information pathways

Of the four genes of the regulatory and information pathways classification, our assembly contradicts the polymorphism upstream of *sigC*, while it corroborates

those in *mazG* and *phoP*. We determined that including the variant in *nrdH* in the set of HC genes by Zheng and colleagues was a mistake, as it is also present in CDC1551.

sigC

Interestingly, the transversion 51bp upstream of *sigC*, contradicted by our assembly, has been cited as the cause of the differential expression of SigC in the E phase and in macrophages relative to H37Rv [2, 3], but it appears there instead must be a distal causative factor [4]. The decreased expression of SigC in macrophage is interesting nonetheless, as its expression is necessary for inducing murine death [5]. Levels of *sigC* mRNA decrease dramatically in response to stress [6], which suggests that increased stress in the macrophage environment in H37Ra may underlie the observed decreased SigC expression in macrophage.

Further background on *phoP*

Chesne-Seck and colleagues uncovered part of the mechanism through which this *phoP* polymorphism may affect the phenotype of H37Ra. They showed that complementation of H37Ra with *phoP* from H37Rv restored synthesis and production of both sulfolipids (SL) and the acyltrehaloses (ATHLs), but not phthiocerol dimycocerosates (PDIMs), all of which are lacking in H37Ra relative to H37Rv. This suggests that the mutation upstream of *phoP* leads to diminished SL and ATHL activity in H37Ra but does not account for the altered activity of PDIMs. Chesne-Seck et al. used a *pks2-3/4* (polyketide synthetase, a necessary precursor of SLs and ATHLs) knock-out H37Rv mutant to assess the effects of the absence of SLs and ATHLs on the virulence of *M. tuberculosis* in mice. The knockout mutants did not exhibit attenuated virulence, which implicates that other mechanisms underlie the effect of *phoP* on virulence attenuation of H37Ra [7].

In a study contrasting expression levels between H37Rv and H37Ra grown in mouse macrophage and nutrient broth, *PhoP* was expressed at higher levels in H37Ra [8]. Of the 50 genes exhibiting differential expression between the two media, 12 were under the regulon of *phoP*. Most interestingly, *ppe18*—one of the 29 contradicted genes, and under the regulon of *phoP*—was underexpressed in H37Ra relative to H37Rv. A potential explanation for this is that PhoP may have not been able to bind to the promoter of *ppe18* due to the SNP-induced aa substitution in the DNA-binding domain of PhoP, which could cause reduced PPE18 expression [7].

The polymorphism in the DNA-binding domain of *phoP* perturbs its autoregulation, altering its expression levels in H37Ra [9, 10]. Though evidence for which direction (positive vs. negative) this point mutation steers the feedback loop in H37Ra is equivocal [9,10], influence in either direction would have downstream effects on all genes under the regulon of *phoP*. Thus, this mutation could affect expression of its regulon both as a result of the change in PhoP's DNA-binding affinity as well as the resulting change in its concentration due to autoregulation.

A recent study by Solans and colleagues [11] provides greater insight into the regulatory role of *phoP* by using ChIP-seq and RNAseq to characterize its regulon. They found that about 2% of the *Mtb* genome is regulated by *phoP*. Thirty genes were bound by *phoP* directly, while an additional 110 had altered expression levels, presumably due to amplification in the signal strength of regulatory cascades initiated by genes under the regulon of *phoP*, such as *espR*, *whiB1*, *whiB3*, and *whiB6*. Interestingly, *lpdA*—found to have a quintuplication of a 58-bp region of its promoter in H37Ra (current study), and differentially expressed in H37Ra relative to H37Rv [4]—appears to be regulated by *phoP*, but not through the known binding motif of *phoP*. The authors propose that an-

other protein may be mediating the interaction between PhoP and the promoter of *lpdA*. Also of great interest is their finding that PhoP-mediated transcription of Mcr7, a small nc-RNA, appears to inhibit translation of TatC through occlusion of its ribosomal binding site. Comparing *phoP* mutants to wild-type, the authors found many putative TAT-secreted substrates to be secreted at significantly higher levels in the *phoP*-mutants. TAT-dependent substrates have been shown to influence immunogenicity. Through RT-qPCR, the authors confirmed that Mcr7 is secreted at lower levels in H37Ra. Considering its effect on secretion of TAT-mediated substrates implicated in immunogenicity, it appears that immunogenic proteins may be excreted at higher levels in H37Ra due to the polymorphism in the DNA-binding site of *phoP*. The levels of ESX-1 proteins under the regulon of *phoP* were unaffected by the differing levels of Mcr7, implying that the two mechanisms of phoP-mediated phenoptypic change are distinct: lack of ESX-1 protein expression leads to diminished synthesis of SL and ATHL, while the reduced expression of Mcr7 may reduce the post-transcriptional repression of Tat proteins, potentially affecting secretion rates of substrates involved in immunogenicity.

ftsH

Our assembly contradicts the transversion and deletion reported upstream of *ftsH*, but this does not alter the picture of what contributes to virulence significantly as it has not, to our knowledge, been implicated in the literature as a virulence factor of any sort.

Cell wall and processes

Our assembly modifies the variant profile of *Rv0039c*, a possible conserved transmembrane protein, and *Rv0037c*, a probable conserved membrane protein. Nei-

ther *Rv0037c* or *Rv0039c* have been implicated as causal to the unique phenotypes of H37Ra, so we cannot speculate further on their involvement.

espK

In H37Ra, *espK* has a SNP that results in an M228I amino acid change, the effect of which may be worth examining. Though *espK* has been characterized as an ESX-1 secretion associated protein, it has not been implicated in the attenuated virulence of H37Ra to our knowledge.

Intermediary metabolism

In the intermediary metabolism functional category, our assembly contradicts polymorphisms in two genes, modifies the variant profile of one gene, and supports all polymorphisms in four genes.

hadC

The recent work by Slama and colleagues is highly important, as it brings the truncating frameshift 1-bp insertion of H37Rv into view as one of the largest contributors to attenuation of virulence in H37Ra compared to H37Rv. It is especially interesting to note that *hadC* was, until quite recently, considered a conserved hypothetical gene [12]. This illustrates the importance of experimentally addressing genes with unknown functions.

lpdA

This repeat region is an experimentally confirmed promoter region of *lpdA*. The additional repeats may alter the affinity with which SigF—a known regulator of LpdA [13]—binds to the promoter of *lpdA*, which modulates the expression of LpdA. *lpdA* was also found to be under the regulon of *phoP*, though through

a regulatory mechanism other than direct binding to the motif for which the polymorphism in *phoP* affects the binding affinity [11]. However, the mutation in *phoP* could still influence LpdA transcription through altered levels of PhoP, due to autoregulatory effects. Though it is unclear whether the differing copy numbers between our assembly and that of H37RV, H37RaJH, and the other H37Rv assemblies were sequencing/assembly errors, or true variation, it is clear that the copy number of this tandem repeat does not underlie the attenuated virulence of H37Ra. The observed differential expression of *lpdA* may be due to altered regulation by PhoP, and may contribute to virulence attenuation—but not due to copy number variation of the 58bp tandem repeat in the promoter of *lpdA*.

ilvD

The contradicted nsSNP in *ilvD* eliminates the only variant directly implicated in amino acid biosynthetic pathways, which suggests that disruption of these pathways does not contribute significantly to the phenotype of H37Ra.

nadD

While the polymorphism in *nadD* was also corroborated and falls in this functional category, it was included erroneously in the HC gene set (Table 1d, Supplementary Table 1). We conclude this because when reproducing Zheng's results, we found that this polymorphism was also present in CDC1551, violating the requirements for the HC gene set [4].

plcD

Our assembly confirms the 8kb deletion in H37Rv relative to H37Ra reported in H37RaJH, which changes the point of disruption of *plcD* from after the 696th bp in H37Rv to after the 476th bp in H37Ra. *plcD* is a known hotspot for IS6110

insertion [14] and strains of H37Rv from other laboratories possess IS6110 in multiple loci in *plcD* [15]. Therefore, it is unlikely that this difference in *plcD* disruption contributes to phenotypic differences in H37Ra.

pabB

We could find no known mechanism of the corroborated promoter-region transversion 66bp upstream of *pabB*, which encodes a precursor to folate biosynthesis, contributing to virulence. However, it is worth noting that H37Ra complemented with *pabB* from H37Rv created significantly larger granulomas in skin lesions of infected rabbits compared to wild-type H37Ra, and that mutations in *pabB* have been shown to attenuate virulence in the bacterium *Burkholderia pseudomallei*, which suggests *pabB* may play a role in virulence yet to be characterized [4, 16].

Lipid Metabolism

pks12

Disruption of *pks12* would dampen PDIM production, which is one of the lipids lacking in H37Ra relative to H37Rv [7, 17] and also deficient after excessive subculturing *in vitro* [18]. In mice, PDIMs have been shown to contribute to growth, and protect tubercle bacilli from reactive nitrogen species in the macrophage environment [19]. PDIM production is not under the influence of *phoP* so, with our refutation of the two SNPs in *phoP*, suggests that there exists a distinct genomic or epigenomic feature causal to the relative lack of PDIM expression in H37Ra [7, 20].

nrp

A nonsense SNP resulting in a truncating stop codon in the *nrp* (non-ribosomal polyketide synthetase) was supported by our assembly. Transposon mutagenesis of the *ppe1-nrp*(Rv0096-Rv0101) operon in *M.bovis* resulted in a strain avirulent in guinea pigs, which suggested *nrp* may play a role in virulence [21, 22].

Additionally, this truncation was thought to potentially affect polyketide synthesis [2]. However, it was later revealed a spontaneous SNP elsewhere in the genome caused the attenuation, implying that mutagenesis of the *ppe1-nrp*(Rv0096-Rv0101) operon did not confer attenuated virulence to *M.bovis* [21–23]. Though this weakens the argument for the *nrp* truncation affecting virulence, it does not necessarily preclude it.

PE_PPE

Specifically, variants in 3 of 15 *pe_pgrs* and 3 of 6 *ppe* genes were supported or adjusted, while the variant in *pe15* was contradicted. While our results distinguish which H37Ra PE_PPE genes are polymorphic with respect to H37Rv, this does not tell the whole story. Of the 5 PE_PPE family members supported or modified by our assembly, all were members of PE_PPE sublineage V. All five were also present in a proteomic sampling *in vivo* of guinea pig lung (using mass spectometry) 90 days following *M.tuberculosis* challenge, of which four were present 30 days following the challenge as well [24, 25]. Though far from conclusive, this points to subfamily V members of the PE_PPE family as potentially more important in virulence than other PE_PPE family members, at least in the case of the differences between H37Rv and H37Ra.

Zhang and colleagues [26] used evolutionary genomics to find genes positively selected for in H37Ra using H37RaJH. They concluded that six genes—four of which were PE_PPE family members—were under positive selection. However,

we contradicted polymorphisms in three of the four (*pe_pgrs22*, *ppe13*, and *pe_pgrs59*) PE_PPE genes and found numerous additional polymorphisms in *pe_pgrs54*.

An *in silico* proteomic study by Kohli and colleagues was also affected greatly by our updated sequence [27], as our assembly alters the amino acid sequence of the majority of PE_PPE genes, which they compared against that of reference sequence H37Rv to make predictions about structural and functional differences between PE_PPE members of the two strains.

Hypothetical and Unknown Genes

Our assembly either modified or supported in full four genes with hypothetical or unkown function. Very little is known about the role the products of these genes may play in virulence, but their variants in H37Ra with respect to H37Rv may be worth exploring. None of the hypothetical or unknown genes for which our assembly contradicted all variants had been implicated in the literature as critical to virulence.

Rv0010c

Interestingly, the corroborated variant with respect to H37Rv which affects *Rv0010c* is an IS6110 element inserted 51bp upstream of the TSS. This is in range to potentially affect transcription through disruption the promoter region for *Rv0010c* by separating it from its TSS.

Rv1006

The supported variant affecting *Rv1006* is a SNP 50bp upstream of its TSS, which could potentially affect its promoter region and, in turn, its expression.

Structural Variants

In addition to the novel structural variants discussed in the main text, our assembly also supported several structural variants reported in H37RaJH.

Our assembly confirmed the presence of RvD2 in H37Ra, an 8kb region deleted in H37Rv which has been determined not to contribute to differences in virulence between H37Ra and H37Rv [28].

Strikingly, the tandem duplication of 1728bp within the coding region of *ppe54* was identical to the 1728bp deletion in *ppe54* of H37RaJH with respect to H37Rv. To illuminate this curiosity, we examined *ppe54* of several *de novo* assembled clinical isolates (unpublished data) of various lineages. Surprisingly, none of the isolates we examined belonging to the Euro-American lineage—the lineage that H37Ra has been assigned to [29]—had this tandem duplication, while many of our East Asian isolates did (in preparation). Further, none of the East Asian isolates with tandem duplications were identical, though they were highly similar (>99% identity), while the tandem duplication in our assembly had identical sequences. In light of this, the deletion in H37RaJH is unlikely to be an assembly error, though we cannot rule out this possibility. If this 1728bp deletion in H37RaJH is correct, then, by our logic, there are two possibilities. One is that the tandem duplication arose in H37Ra in culture prior to the last common ancestor of the culture from which H37RaJH was derived and ours, and both copies of the 1728bp region were deleted in the culture sequenced by Zheng and colleagues [4]. The second possibility is that the most recent common ancestor of H37RaJH and our strain had one copy of the 1728bp region, and both the deletion in H37RaJH and the tandem duplication in our assembly occurred after they diverged from one another. The 100% identity between each 1728bp duplicate of the tandem repeat suggests that the latter scenario is more likely.

References

[1] Lew, J.M., Kapopoulou, A., Jones, L.M., Cole, S.T.: TubercuList – 10 years after. Tuberculosis **91**(1), 1–7 (2011). doi:10.1016/j.tube.2010.09.008

[2] Dokladda, K., Billamas, P., Palittapongarnpim, P.: Different behaviours of promoters in *Mycobacterium tuberculosis* H37Rv and H37Ra. World Journal of Microbiology and Biotechnology **31**(2), 407–413 (2015). doi:10.1007/s11274-014-1794-x

[3] Malhotra, V., Tyagi, J.S., Clark-Curtiss, J.E.: DevR-mediated adaptive response in *Mycobacterium tuberculosis* H37Ra: links to asparagine metabolism. Tuberculosis (Edinburgh, Scotland) **89**(2), 169–174 (2009). doi:10.1016/j.tube.2008.12.003

[4] Zheng, H., Lu, L., Wang, B., Pu, S., Zhang, X., Zhu, G., Shi, W., Zhang, L., Wang, H., Wang, S., Zhao, G., Zhang, Y.: Genetic basis of virulence attenuation revealed by comparative genomic analysis of *Mycobacterium tuberculosis* strain H37Ra versus H37Rv. PLoS One **3**(6), 2375 (2008). doi:10.1371/journal.pone.0002375

[5] Sun, R., Converse, P.J., Ko, C., Tyagi, S., Morrison, N.E., Bishai, W.R.: Mycobacterium tuberculosis ECF sigma factor sigC is required for lethality in mice and for the conditional expression of a defined gene set. Molecular Microbiology **52**(1), 25–38 (2004). doi:10.1111/j.1365-2958.2003.03958.x

[6] Manganelli, R., Dubnau, E., Tyagi, S., Kramer, F.R., Smith, I.: Differential expression of 10 sigma factor genes in Mycobacterium tuberculosis. Molecular Microbiology **31**(2), 715–724 (1999). doi:10.1046/j.1365-2958.1999.01212.x

[7] Chesne-Seck, M.-L., Barilone, N., Boudou, F., Asensio, J.G., Kolattukudy, P.E., Martín, C., Cole, S.T., Gicquel, B., Gopaul, D.N., Jackson, M.: A point mutation in the two-component regulator PhoP-PhoR accounts for the absence of polyketide-derived acyltrehaloses but not that of phthiocerol dimycocerosates in *Mycobacterium tuberculosis* H37Ra. Journal of Bacteriology **190**(4), 1329–1334 (2008). doi:10.1128/JB.01465-07

[8] Li, A.H., Waddell, S.J., Hinds, J., Malloff, C.A., Bains, M., Hancock, R.E., Lam, W.L., Butcher, P.D., Stokes, R.W.: Contrasting transcriptional responses of a virulent and an attenuated strain of *Mycobacterium tuberculosis* infecting macrophages. PLoS One **5**(6) (2010). doi:10.1371/journal.pone.0011066

[9] Gonzalo-Asensio, J., Soto, C.Y., Arbués, A., Sancho, J., Menéndez, M.d.C., García, M.J., Gicquel, B., Martín, C.: The Mycobacterium tuberculosis phoPR Operon Is Positively Autoregulated in the Virulent Strain H37Rv. Journal of Bacteriology **190**(21), 7068–7078 (2008). doi:10.1128/JB.00712-08

[10] Gupta, S., Sinha, A., Sarkar, D.: Transcriptional autoregulation by Mycobacterium tuberculosis PhoP involves recognition of novel direct repeat sequences in the regulatory region of the promoter. FEBS Letters **580**(22), 5328–5338 (2006). doi:10.1016/j.febslet.2006.09.004

[11] Solans, L., Gonzalo-Asensio, J., Sala, C., Benjak, A., Uplekar, S., Rougemont, J., Guilhot, C., Malaga, W., Martín, C., Cole, S.T.: The PhoP-dependent ncRNA Mcr7 modulates the TAT secretion system in *Mycobacterium tuberculosis*. PLoS Pathogens **10**(5) (2014). doi:10.1371/journal.ppat.1004183

[12] Slama, N., Jamet, S., Frigui, W., Pawlik, A., Bottai, D., Laval, F., Constant, P., Lemassu, A., Cam, K., Daffé, M., Brosch, R., Eynard, N., Quémard, A.: The changes in mycolic acid structures caused by hadC mutation have a dramatic effect on the virulence of *Mycobacterium tuberculosis*. Molecular Microbiology, (2015). doi:10.1111/mmi.13266

[13] Akhtar, P., Singh, S., Bifani, P., Kaur, S., Srivastava, B.S., Srivastava, R.: Variable-number tandem repeat 3690 polymorphism in Indian clinical isolates of *Mycobacterium tuberculosis* and its influence on transcription. Journal of Medical Microbiology **58**(6), 798–805 (2009). doi:10.1099/jmm.0.002550-0

[14] Vera-Cabrera, L., Hernández-Vera, M.A., Welsh, O., Johnson, W.M., Castro-Garza, J.: Phospholipase Region of Mycobacterium tuberculosis Is a Preferential Locus for IS6110 Transposition. Journal of Clinical Microbiology **39**(10), 3499–3504 (2001). doi:10.1128/JCM.39.10.3499-3504.2001

[15] Ioerger, T.R., Feng, Y., Ganesula, K., Chen, X., Dobos, K.M., Fortune, S., Jacobs, W.R., Mizrahi, V., Parish, T., Rubin, E., Sassetti, C., Sacchettini, J.C.: Variation among genome sequences of H37Rv strains of *Mycobacterium tuberculosis* from multiple laboratories. Journal of Bacteriology **192**(14), 3645–3653 (2010). doi:10.1128/JB.00166-10

[16] Zhang, G., Zhu, B., Shi, W., Wang, M., Da, Z., Zhang, Y.: Evaluation of mycobacterial virulence using rabbit skin liquefaction model. Virulence **1**(3), 156–163 (2010). doi:10.4161/viru.1.3.11748

[17] Sirakova, T.D., Dubey, V.S., Kim, H.-J., Cynamon, M.H., Kolattukudy, P.E.: The Largest Open Reading Frame (pks12) in the Mycobacterium tuberculosis Genome Is Involved in Pathogenesis and Dimycocerosyl Ph-

thiocerol Synthesis. Infection and Immunity **71**(7), 3794–3801 (2003). doi:10.1128/IAI.71.7.3794-3801.2003

[18] Andreu, N., Gibert, I.: Cell population heterogeneity in *Mycobacterium tuberculosis* H37Rv. Tuberculosis (Edinburgh, Scotland) **88**(6), 553–559 (2008). doi:10.1016/j.tube.2008.03.005

[19] Rousseau, C., Winter, N., Pivert, E., Bordat, Y., Neyrolles, O., Avé, P., Huerre, M., Gicquel, B., Jackson, M.: Production of phthiocerol dimycocerosates protects *Mycobacterium tuberculosis* from the cidal activity of reactive nitrogen intermediates produced by macrophages and modulates the early immune response to infection. Cellular Microbiology **6**(3), 277–287 (2004). doi:10.1046/j.1462-5822.2004.00368.x

[20] Asensio, J.G., Maia, C., Ferrer, N.L., Barilone, N., Laval, F., Soto, C.Y., Winter, N., Daffé, M., Gicquel, B., Martín, C., Jackson, M.: The virulence-associated two-component PhoP-PhoR system controls the biosynthesis of polyketide-derived lipids in *Mycobacterium tuberculosis*. Journal of Biological Chemistry **281**(3), 1313–1316 (2006). doi:10.1074/jbc.C500388200

[21] Hotter, G.S., Wards, B.J., Mouat, P., Besra, G.S., Gomes, J., Singh, M., Bassett, S., Kawakami, P., Wheeler, P.R., de Lisle, G.W., Collins, D.M.: Transposon mutagenesis of Mb0100 at the *ppe1-nrp* locus in *Mycobacterium bovis* disrupts phthiocerol dimycocerosate (PDIM) and glycosylphenol-PDIM biosynthesis, producing an avirulent strain with vaccine properties at least equal to those of *M. bovis* BCG. Journal of Bacteriology **187**(7), 2267–2277 (2005). doi:10.1128/JB.187.7.2267-2277.2005

[22] Hotter, G.S., Collins, D.M.: *Mycobacterium bovis* lipids: Virulence and vaccines. Veterinary Microbiology **151**(1–2), 91–98 (2011). doi:10.1016/j.vetmic.2011.02.030

[23] Köser, C.U., Niemann, S., Summers, D.K., Archer, J.A.C.: Overview of errors in the reference sequence and annotation of *Mycobacterium tuberculosis* H37Rv, and variation amongst its isolates. Infection, Genetics and Evolution **12**(4), 807–810 (2012). doi:10.1016/j.meegid.2011.06.011

[24] Kruh, N.A., Troudt, J., Izzo, A., Prenni, J., Dobos, K.M.: Portrait of a Pathogen: The Mycobacterium tuberculosis Proteome In Vivo. PLoS One **5**(11) (2010). doi:10.1371/journal.pone.0013938

[25] Fishbein, S., van Wyk, N., Warren, R.M., Sampson, S.L.: Phylogeny to function: PE/PPE protein evolution and impact on *Mycobacterium tuberculosis* pathogenicity. Molecular Microbiology **96**(5), 901–916 (2015). doi:10.1111/mmi.12981

[26] Zhang, Y., Zhang, H., Zhou, T., Zhong, Y., Jin, Q.: Genes under positive selection in *Mycobacterium tuberculosis*. Computational Biology and Chemistry **35**(5), 319–322 (2011). doi:10.1016/j.compbiolchem.2011.08.001

[27] Kohli, S., Singh, Y., Sharma, K., Mittal, A., Ehtesham, N.Z., Hasnain, S.E.: Comparative genomic and proteomic analyses of PE/PPE multigene family of *Mycobacterium tuberculosis* H37Rv and H37Ra reveal novel and interesting differences with implications in virulence. Nucleic Acids Research **40**(15), 7113–7122 (2012). doi:10.1093/nar/gks465

[28] Lari, N., Rindi, L., Garzelli, C.: Identification of one insertion site of IS6110 in *Mycobacterium tuberculosis* H37Ra and analysis of the RvD2 deletion in *M. tuberculosis* clinical isolates. Journal of Medical Microbiology **50**(9), 805–811 (2001). doi:10.1099/0022-1317-50-9-805

[29] Gagneux, S., Small, P.M.: Global phylogeography of Mycobacterium tuberculosis and implications for tuberculosis product development. The

Lancet. Infectious Diseases **7**(5), 328–337 (2007). doi:10.1016/S1473-3099(07)70108-1

Table 1: Functional category and confirmation status of genes previously reported as affected by H37Ra-specific mutations

Status	Functional Category (Count)	Gene
Contradicted	lipid metabolism (1)	Rv2048c
	information pathways (1)	Rv2069
	cell wall and cell wall processes (2)	Rv0037c<br>Rv0383c
	insertion seqs and phages (2)	Rv2649<br>Rv3191c
	PE/PPE (16)	Rv0124<br>Rv0279c<br>Rv0578c<br>Rv0977<br>Rv1068c<br>Rv1091<br>Rv1196<br>Rv1386<br>Rv1450c<br>Rv1802<br>Rv2098c<br>Rv2396<br>Rv3350c<br>Rv3388<br>Rv3507<br>Rv3595c
	intermediary metabolism and respiration (6)	Rv0189c<br>Rv1095<br>Rv2068c<br>Rv2202c<br>Rv3303c<br>Rv3389c
	regulatory proteins (1)	Rv0880
	conserved hypotheticals (7)	Rv1929c<br>Rv2733c<br>Rv2734<br>Rv2825c<br>Rv3031<br>Rv3192<br>Rv3611
Adjusted	insertion seqs and phages (1)	Rv1764
	PE/PPE (3)	Rv3343c<br>Rv3508<br>Rv3514
Supported	lipid metabolism (1)	Rv0101
	cell wall and cell wall processes (3)	Rv0010c<br>Rv0039c<br>Rv3879c
	PE/PPE (3)	Rv0878c<br>Rv1759c<br>Rv2352c
	intermediary metabolism and respiration (4)	Rv0635<br>Rv0637<br>Rv1005c<br>Rv1755c
	regulatory proteins (1)	Rv0757
	conserved hypotheticals (2)	Rv1006<br>Rv1021
Erroneous	information pathways (1)	Rv3053c
	intermediary metabolism and respiration (1)	Rv2421c

Genes originally reported to be affected by H37Ra-specific mutations [4] were placed into the four groups "contradicted", "adjusted", "supported", and "erroneous" based on replicative analysis and comparisons with our assembly of the H37Ra genome (Main text: Results, Table 1). The genes in each of these four groups are further categorized here by their TubercuList functional categories [1].
